# The “Ledoyen Disinfecting Fluid”

**Published:** 1848-01

**Authors:** 


### THE "LEDOYEN DISINFECTING FLUID."

For some time past, the medical journals of Great Britain have been animadverting on the encouragement given to empiricism by the British Government, in directing experiments to be instituted for disinfecting the subjects of contagious diseases, as well as infected localities, with a preparation suggested by M. Ledoyen, which is offered for sale in our shops, and has acquired no little notoriety. We have not examined it, but it is said to be capable of removing offensive odours, at least as effectually as any other agent. The mistake was, however, incurred in supposing—as has been over and over again supposed, and as often negatived—that if offensive odours are destroyed, contagious and other miasmata will share the like fate. It is not many years since Professor Daniel, of London, affirmed, that the essence of the malaria of the Niger is sulphuretted hydrogen; and as sulphuretted hydrogen is decomposed by chlorine, he inferred, that it is but necessary for a ship sailing in the waters of the Niger to be provided with an apparatus that can disengage a sufficient quantity of chlorine, and then it may remain in those pestilential localities with impunity. Observation showed not only fallacy of fact, but fallacy of argument. The sulphuretted hydrogen was proved not to exist in the waters of the Niger, but to be the product of the decomposition of the specimens sent to England. Ships were liberally furnished by the government with expensive apparatuses for the disengagement of the gas; and after they had been thus furnished they suffered more disastrously from the endemic of the country than they had ever done previously. The chlorine was found to possess no disinfecting power over the malaria.

In the same disastrous manner has the Ledoyen "disinfection" eventuated. In the *British and American Journal of Medical and Physical Science*, for December, 1847, we learn that Col. Calvert—the great supporter of M. Ledoyen, and asserter of the powerful disinfecting virtues of the Ledoyen fluid—has died a victim to the disease, to neutralize the contagious property of which he visited this continent in company with its inventor.

"At Quebec, on the 12th of November, of typhus, Colonel Calvert, who accompanied M. Ledoyen to this country, under the authority of the British Government, to test the efficacy of the disinfecting fluid proposed by the latter as a preventive of the spread of typhus, by destroying the contagious miasm. The commonest justice to Col. Calvert requires us to record our sincere conviction of his thorough belief in the efficacy of M. Ledoyen's agent, and in his falling a victim himself to typhus fever in his sedulous endeavours to demonstrate the benefits obtainable from the employment of the fluid, he has been, according to the inscrutable ways of Providence, permitted to exhibit, in his own person, the utter futility of the means which he himself advocated so strenuously."

It would appear from the following article in the same journal, that experiments have been made in the Canadas, both with the fluid proposed by M. Ledoyen, and with that suggested by Sir Wm. Burnett, as a disinfecting and antiseptic agent. The active material in the former is said to be nitrate of lead; in the latter, chloride of zinc, which, as an antiseptic, in the dissecting room, has the testimonials of Messrs. Bowman, Sharpey and others, in its favour. (See Dunglison's *New Remedies*, p. 600, edit. of 1846.)

*The Disinfecting Fluids.*—The experiments with these fluids have been brought to a close, and from all that we have heard and read upon the subject, our opinion as to any *disinfecting* properties possessed by either Sir W. Burnett's or M. Ledoyen's is still unaltered. According to the results of some experiments made at the Marine Hospital, Quebec, to determine which possessed the greater power in mitigating or destroying the effluvium from soil, votes were given in favour of Sir W. Burnett's fluid. We apprehend, however, that not much difference exists between them both in this respect. Some disagreement having arisen between the experimenters, M. Ledoyen treated the profession in Quebec to some novel therapeutic ideas in relation to the injurious agency of the preparations of zinc on the animal economy when applied to, and absorbed from, ulcerated surfaces. This is worth noticing, only in so far as it evinces to what extent a preconceived notion, with strong enthusiasm, can warp the judgment and influence the reasoning faculties of an individual. M. Ledoyen has left for England, after having suffered from typhus himself; and poor Colonel Calvert is no more, having succumbed to a more virulent attack of the same disease. We sincerely sympathize with Colonel Calvert's family in the bereavement which they have suffered; but, at the same time, we regard the consequences to M. Ledoyen and Colonel Calvert as a strong proof of the fallacy of the views which they entertained, and as affording matter for a homily on the whole affair.

